# Detailed analysis of cystic lesions in patients after open fetal repair and postnatal myelomeningocele closure

**DOI:** 10.1007/s00381-024-06735-8

**Published:** 2025-01-03

**Authors:** Sanjana R. Salwi, Sierra D. Land, Taryn Gallagher, Tom A. Reynolds, Deborah M. Zarnow, Angela Viaene, Julie S. Moldenhauer, N. Scott Adzick, Tracy M. Flanders, Gregory G. Heuer

**Affiliations:** 1 Division of Neurosurgery, Department of Surgery, Children’s Hospital of Philadelphia, Philadelphia, PA USA; 2 Richard D. Wood Jr. Center for Fetal Diagnosis and Treatment, Children’s Hospital of Philadelphia, Philadelphia, PA USA; 3 Department of Radiology, Children’s Hospital of Philadelphia, Philadelphia, PA USA; 4 Division of Anatomic Pathology, Children’s Hospital of Philadelphia, Philadelphia, PA USA

**Keywords:** Fetal closure, Myelomeningocele, Inclusion cyst, Tethered cord

## Abstract

**Purpose:**

We sought to evaluate the incidence, natural history, and management of cystic spinal lesions following myelomeningocele/myeloschisis closure.

**Methods:**

We performed a single-center retrospective review of all patients who underwent myelomeningocele/myeloschisis closure from 2013 to 2018 with follow-up to 5 years old.

**Results:**

We analyzed 100 fetal repairs and 81 postnatal closures from 305 total surgeries. Patients within this cohort systematically underwent serial MRI scans of the lumbar spine and had clinical follow-up until at least 5 years of age. Ninety-three (51.2%) developed radiographic evidence of cystic lesions with 28 (30.1%) requiring surgical intervention. Presence of cysts was higher in fetal repair (67/100, 67%) compared with postnatal (26/81, 32.1%; *p* < 0.01). Of the 93 patients with radiographic cysts, 28 (30.1%) underwent surgical resection at a median age of 27.6 months old ([Q1, Q3], [13.0, 48.6 months]). Fetal repair patients had a higher rate (26/67, 38.8%) of cysts requiring surgical resection compared with postnatal closure (2/26, 7.7%, *p* value < 0.01). Pathology demonstrated 18 of resected cysts were dermoid, 8 were epidermoid, and 2 were fibrous tissue. Post-operatively, no patients experienced a worsened ambulation status. Bladder compliance showed a non-significant trend toward improvement.

**Conclusions:**

Cystic lesions in myelomeningocele/myeloschisis patients are common findings that result in nerve root tethering. We propose regular screening in both symptomatic and asymptomatic patients to circumvent nerve injury. Most cystic lesions do not require surgical resection though fetal repair is associated with a higher incidence of operative cysts. However, these lesions can be safely surgically resected with maintenance of ambulation and urologic function.

## Introduction

Myelomeningocele (MMC) and myeloschisis are the most common and most severe forms of open spina bifida with lifelong functional implications [1]. Even after initial fetal repair or postnatal closure of the neural tube defect, patients will often require multiple surgical procedures for correction of pathologic sequelae including hydrocephalus, Chiari II, tethered cord syndrome (TCS), and various urologic or orthopedic issues [2, 3]. Improved monitoring and treatment of these complications, especially the development of TCS, is paramount to improving quality of life and increasing life expectancy for these patients [4, 5]. Spinal cysts are a heterogenous entity, some cyst can be composed of epidermoid or dermoid tissue [6]. They occur in patients after both fetal repair and postnatal closure, though they have been shown to occur at higher rates in patients that have undergone fetal repair [7–10]. It is unclear how these cysts contribute to the pathophysiology of TCS and affect long-term outcomes of patients after MMC/myeloschisis repair [11].

Given their rarity, the overall rate of formation, natural history, and best management of these spinal cysts are not well-established. Herein, we present the largest case series to date of patients that underwent open fetal closure or postnatal repair and then ultimately developed spinal cystic lesions.

## Methods

We retrospectively reviewed consecutive cases of open fetal repair or postnatal closure of MMC/myeloschisis at a single institution between January 1, 2013, and December 31, 2018.

Patients with long-term follow up (clinic visit (in-person or telehealth) and radiographic follow-up with spinal magnetic resonance imaging (MRI) until at least 5 years of age) were included. Per our institutional surveillance imaging protocol, follow-up brain and spine MRIs are obtained at birth, 6 months, 1 year, and lumbar spine MRI annually thereafter. The Clinical Outcomes Data Archive (CODA) provided demographic information.

The following variables were collected using the institution’s electronic medical record (EMR) system. Baseline demographic variables included anatomic lesion level (defined as osseous level on prenatal ultrasound), presence of talipes (yes/no), need for CSF diversion (yes/no), total defect area (measured intra-op, cm^2^), type of surgical intervention (fetal repair vs. postnatal closure), presence of (yes/no), and highest extent of syrinx (defined by region: cervical, thoracic, lumbar, vs. sacral). The main variables of interest were radiographic presence of spinal cyst (defined as cyst found on any follow-up spine MR) and surgical resection of cyst. Additionally, age at cyst resection and surgical indication (symptoms and/or radiographic progression) were included. Symptoms for surgical indication were assessed by physical examination, urodynamic testing, and/or clinical history provided by parent. The primary outcome measure described for the surgical cohort was ambulation status defined as non-ambulator, ambulation with assistance devices, and independent ambulation. Secondary outcomes included radiographic recurrence of cyst, concurrently diagnosed hydrocephalus requiring CSF diversion, final pathology, and urologic function. Bladder function was measured qualitatively with urinary management strategy (yes/no need for clean intermittent catheterization (CIC)) and quantitatively by measuring bladder compliance (pressure at actual bladder capacity) using a VUDS (video urodynamic study).

### Statistical analysis

Descriptive statistics were reported as frequencies for categorical variables or median with interquartile range (IQR) and mean with standard deviation (SD) for continuous variables. The association between outcome variables was assessed using Chi-square, and fisher’s exact test for categorical variables and Student’s *t*-test or Mann–Whitney *U*-test as appropriate for continuous variables. All statistical analyses and figures were performed with R Statistical Software (v4.1.2; R Core Team 2021).

## Results

### Patient characteristics

**Fig. 1:**
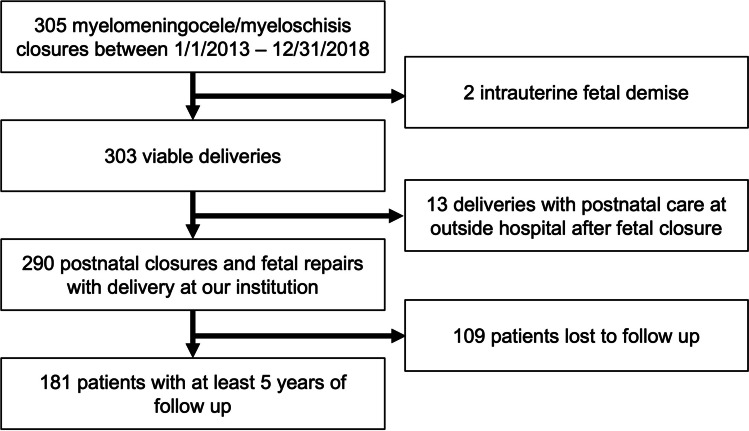
Flow chart showing inclusion and exclusion criteria applied to create final cohort of patients

A total of 305 consecutive patients underwent open fetal repair or postnatal closure for MMC/myeloschisis between January 1, 2013, and December 31, 2018. Among the patients who underwent fetal repair, 2 patients were excluded secondary to intrauterine fetal demise, and 13 were excluded for delivering and receiving postnatal care at a different hospital. Within both populations, 109 patients were lost to follow-up leading to a final cohort of 181 patients (Fig. 1).

In total, 862 MRIs were evaluated for our cohort with a mean (SD) of 4.8 (3.5) scans per patient. And 133 patients (73.5%) had at least 1 MRI per year of follow-up with the remainder having at least one MRI at 5 years of age. The mean age at the last clinical follow up was 6.5 years old (SD 2.7, median 6.0; range 4.5–7.5). Then, 93 (51.2%) patients had a spinal cyst found on MRI. In comparing patients without and with radiographic evidence of cyst, there was no difference in proportion of patients with a diagnosis of myelomeningocele (no cyst = 73/88 (83.0%), cyst = 67/93 (72.0%), *p* value = 0.115), presence of pre-natal talipes (no cyst = 20/88 (22.7%), cyst = 18/93 (19.4%), *p* value = 0.708), and need for CSF diversion (no cyst = 53/88 (60.2%), cyst = 41/93 (44.1%), *p* value = 0.043). There was also no difference in defect area (median, [IQR], cm^2^) for patients with no cyst (9.0 [6.0, 16.0]) and with cyst (7.5 [5.5, 12.0], *p* value = 0.164). A larger percentage of patients without cysts had an osseous defect of L4 and below (47/88, 53.4%) compared with those with cysts (36/93, 38.7%, *p* value = 0.045). There was a significantly higher rate of cysts after fetal repair (67/100, 67%) when compared with postnatal (26/81, 32.1%) closure (*p* value < 0.001). These baseline demographics are summarized in Table 1.

**Table 1:** Baseline characteristics of patients with and without radiographic spinal cysts

	No spinal cyst (*N* = 88)	Spinal cyst (*N* = 93)	*p* Value
Diagnosis			
Myelomeningocele	73 (83.0%)	67 (72.0%)	0.115
Myeloschisis	15 (17.0%)	26 (28.0%)	
Prenatal talipes			
Yes	20 (22.7%)	18 (19.4%)	0.708
CSF diversion			
Yes	53 (60.2%)	41 (44.1%)	0.043
Defect area (surgery)			
Median [Q1, Q3]	9.00 [6.0, 16.0]	7.50 [5.5, 12.0]	0.164
Mean (SD)	13.7 (13.2)	11.37 (11.1)	
Missing	21 (23.9%)	22 (23.7%)	
Lesion level			
T12 and above	1 (1.1%)	6 (6.5%)	0.045
L1–L3	40 (45.5%)	51 (54.8%)	
L4 and below	47 (53.4%)	36 (38.7%)	
Repair technique			
Fetal	33 (37.5%)	67 (72.0%)	< 0.001
Postnatal	55 (62.5%)	26 (28.0%)	

### Surgical cohort descriptive variables

**Fig. 2:**
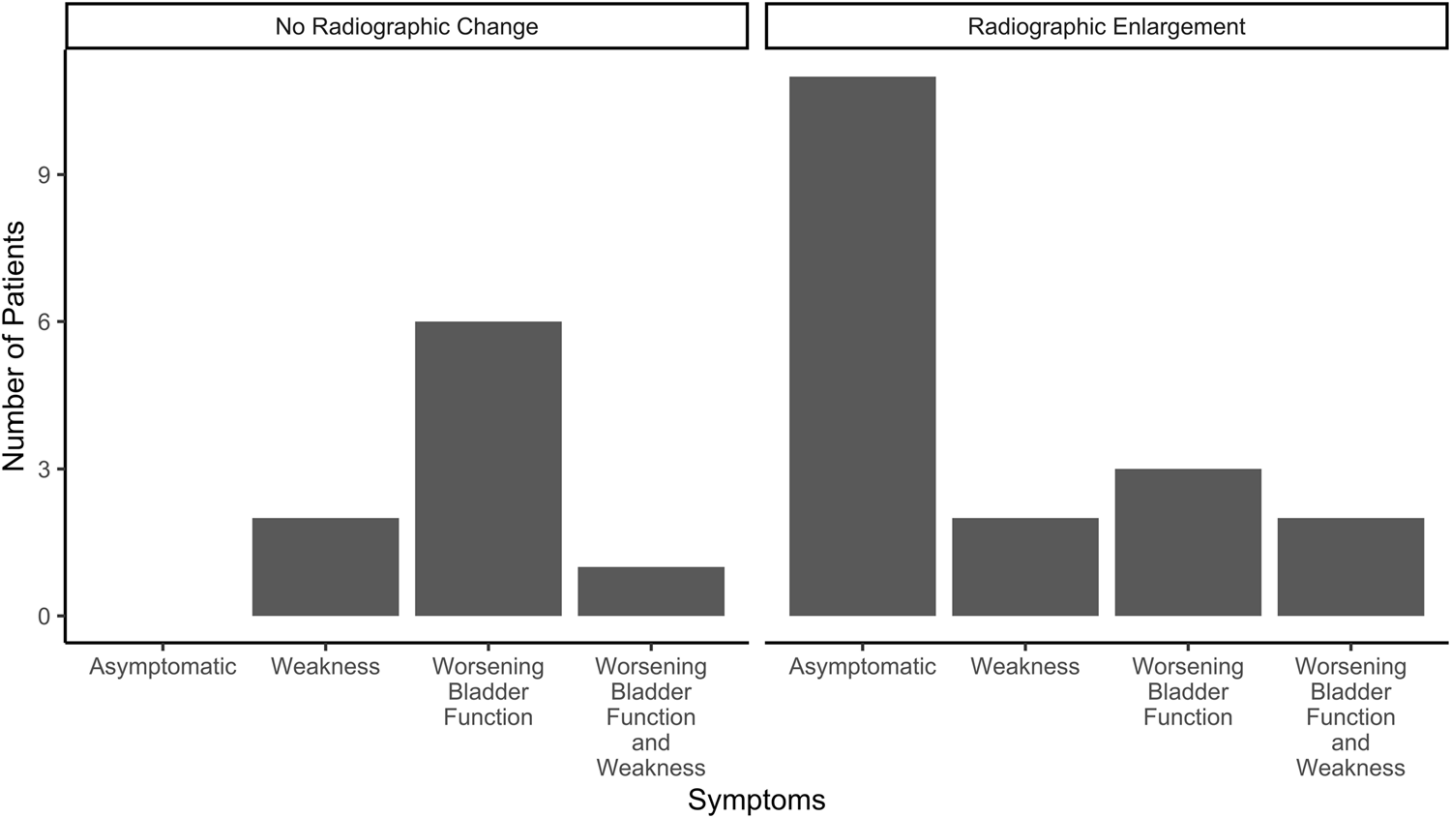
Counts of patients for each surgical indications separated by those with and without radiographic evidence of spinal cyst

Of the 93 patients with radiographic cysts, 28 (30.1%) underwent surgical resection. Patients that underwent fetal repair had a higher rate of requiring surgical resection (26/67, 38.8%) compared with postnatal closure (2/26, 7.7%, *p* value = 0.007). Surgical indications included development of symptoms and/or radiographic enlargement of cyst. Of the patients with radiographic enlargement of cyst, 11 (39.3%) were asymptomatic. Of the patients with radiographic enlargement and symptoms, two patients presented with weakness, three patients with worsening bladder function, and two patients had both. Nine patients had symptoms (weakness = 2, worsening bladder function = 6, both = 1) without radiographic change. These surgical indications are summarized in Fig. 2. At the time of resection, patients were a median age of 27.6 months old ([Q1, Q3], [13.0, 48.6 months], mean (SD) = 32.1 (22.2) months). Surgical resection occurred at a mean of 15.6 months (SD = 18.1, median [Q1, Q3] = 7.6 [2.5, 21.2 months]) after cyst was first found on imaging.

### Outcomes of surgical cohort

No patients had worsened ambulation status following surgical resection. Two patients had a developmentally expected improvement in their ambulation status. They were previously unable to walk due to age. At their postoperative follow-up, one ambulated independently and one with assistive devices (Table 2).

**Table 2:** Baseline demographics for resection of spinal cysts

	No surgical resection (*n* = 65)	Surgical resection (*n* = 28)	*p* Value
	*n* (%)	*n* (%)	
Repair technique			
Fetal	41 (63.1)	26 (92.9)	0.007
Postnatal	24 (36.9)	2 (7.1)	
Presence of syrinx			
Yes	30 (46.2)	10 (35.7)	0.481
Highest extent of syrinx			
Cervical	3 (4.6)	1 (3.6)	0.872
Thoracic	13 (20.0)	3 (10.7)	
Lumbar	12 (18.5)	5 (17.9)	
Sacral	2 (3.1)	1 (3.6)	

Of the surgical cohort of 28 patients, 16 patients (57.1%) had radiographic recurrence of cyst on surveillance imaging. Five patients (17.9%) in the surgical cohort had concurrently enlarged ventricles on preoperative imaging and had an external ventricular drain placed intra-operatively to evaluate for need for CSF diversion. Postoperatively, these patients met criteria for clinical hydrocephalus and the external ventricular drain was converted into a ventriculoperitoneal shunt. Final pathology review of surgical specimens showed that the majority were dermoid cysts (18/28, 64.3%), 8 were epidermoid cysts (28.6%), and 2 were fibrous tissue (7.1%).

Twenty-four patients had close urologic follow-up prior to surgery (median = 2.8 months [Q1, Q3] [1.3 months, 3.9 months] and post-operatively (median = 6.9 months [Q1, Q3] [6.3 months, 7.6 months]). No patients had worsened bladder function that required a new clean intermittent catheterization (CIC) protocol after surgery. Twenty patients (83.4%) remained stable in their urinary management strategy, and 4 patients (16.7%) had improvement postoperatively with no further need for CIC. Fourteen of the 20 patients also had pre/postoperative VUDS performed to assess bladder function. Of these, 8 (57.1%) had no significant difference in bladder compliance measured by pressure at actual bladder capacity (cmH_2_0) (before, median [Q1, Q3], 11.0 [3.25, 20.75] vs. after 9.0 [5.0, 18.75]; *p* value = 0.73). Two patients (14.3%) had improved bladder capacity, while four patients had worsened bladder capacity (28.6%). These results are summarized in Table 3.

**Table 3:** Functional outcomes after surgical resection. *CIC* clean intermittent catheterization

	Overall
	(*n* = 24)
Duration from pre-assessment to resection (months)	
Median [Q1, Q3]	2.75 [1. 27, 3.88]
Mean (SD)	3.88 (3.87)
Duration from resection to post-assessment (months)	
Median [Q1, Q3]	6.90 [6.30, 7.60]
Mean (SD)	7.33 (2.02)
Change in ambulation status	
No change	22 (91.6%)
Improvement	2 (8.3%)
Change in urinary management	
No change: using CIC pre + post	7 (29.2%)
No change: no CIC pre + post	13 (54.2%)
Improvement: stopped CIC post resection	4 (16.7%)

## Discussion

Given that it can take years for cysts to develop, [10] our robust 5-year follow-up with interval imaging of symptomatic and asymptomatic patients allows us to present long-term functional and safety outcomes. At our institution, we obtain spinal imaging for patients after neurosurgical intervention for open spina bifida at birth, 6 months, 1 year, and annually thereafter. Though many protocols can describe ideal surveillance imaging, our results benefit from demonstrated thorough follow-up. All patients were followed with MRI and clinical follow-up at some interval until at least 5 years of age, and three-fourths of patients received at least yearly MRIs regardless of symptoms. Therefore, we can report the rate of radiographic spinal cyst formation in asymptomatic patients after both fetal and postnatal repair. In comparison, prior studies report on only incidence of spinal cysts and outcomes of symptomatic patients [9].

The other major study that reported on asymptomatic spinal cysts was also performed at our institution and reported outcomes of 54 patients that underwent fetal repair prior to the Management of Myelomeningocele Study (MOMS) trial between the years of 1998 and 2003 [8]. The majority (63%) did not have any clinical or radiographic evidence of cyst. Ten (18.5% compared to 30% in our study) patients required surgical intervention for symptomatic TCS with cyst, while 4 (7.4%) were asymptomatic with radiographic evidence of cyst. One (10%) patient had deterioration in ambulatory status, and four (40%) had worsened urologic function requiring CIC. It is unknown if this decline is related to the surgery or because of the cyst itself.

In the larger and more contemporary cohort represented in this study, we show a higher rate of cyst formation in patients after fetal repair (67%) compared with the rates reported by Flanders et al. (33.6%) [12] and Danzer et al. (26%) [7]. Another recent study looking at allograft patch closure and formation of cysts showed a rate of 23% of spinal inclusion cyst formation. However, planned interval imaging for all patients only occurred for 3 years. The higher rate of cysts seen on MRI in our study is likely related to the robust surveillance imaging on all patients and longer-term follow up, both of which allowed us to identify cysts that developed at older ages. Prior to the implementation of our current surveillance imaging protocol, patients would get MRI at birth, at 1 year, and then only if symptoms developed. This study benefits from a longer and more robust screening protocol as well as achievement of the surveillance goal. Also, the current study is a more complete assessment of all cystic lesions on the MRI and not just inclusion cyst formation. Our findings suggest that more patients than previously thought develop cysts after MMC repair, though most remain asymptomatic. It behooves us as long-term care providers for these patients to proactively discern these spinal inclusion cysts prior to the development of irreversible neurologic compromise. Though our more stringent and frequent serial imaging technique is more extensive, this diligence confers better care for these patients as tethering can be radiographically detected.

At present, the etiology of cyst lesions and more specifically inclusion cysts after MMC/myeloschisis intervention in both the prenatal and postnatal periods is unknown. One theory conjectures that these cysts are an iatrogenic consequence of in utero repair secondary to inadvertent inclusion of the dermal layer during closure. Another hypothesis suggests that closure with acellular dermis graft acts as scaffolding for subsequent cyst formation [9, 13]. Another theory speculates that these cysts are a result of an index dysembryogenesis pattern secondary to cutaneous ectodermal rests pathologically sequestered in the dysplastic spinal cord during secondary neurulation rather than from the closure itself [14, 15]. Patients can develop these cysts even after postnatal repair supporting the idea of an index dysembryogenesis event that can cause later onset neurologic decline requiring surgical intervention [14, 16].

One-third of our postnatal MMC repair patients showed radiographic evidence of cysts, lending credence to the last theory. Two of these patients required surgical intervention. While prior work has shown the presence of inclusion cysts after postnatal repair, the question has remained if the higher incidence of inclusion cysts after fetal repair was secondary to more robust screening of these patients [13]. We show that when both populations undergo regular yearly monitoring, there is a true higher incidence of spinal cysts in patients after fetal repair (67% fetal vs. 32% postnatal). These rates are important for prenatal counseling. Additionally, given the known association with spinal cysts and neurological decline, our data show a role for surveillance imaging for postnatal repair patients to identify patients that require close follow-up and surgical resection prior to or as soon as possible with the development of neurologic changes [17, 18]. In addition, qualitatively cysts that are smaller and have not incorporated nerve roots are more easily resected. While there was a recurrence of some cysts in this study, great attention of resection of the cyst wall along with the cystic contains has resulted in a reduction in this rate.

Eight asymptomatic patients underwent surgical resection of a cyst. These patients maintained their baseline urologic and neurologic function after resection and have remained asymptomatic through the 5-year total follow-up. Though longer-term follow up is needed to see recurrence rates and need for surgical re-intervention, all the patients in our surgical cohort maintained or improved their preoperative ambulatory status. In contrast to our previously reported cohort, no patients had a decline in bladder function that required a new CIC protocol postoperatively [12]. This finding is in line with prior work showing nonworsening of urologic function after tethered cord release in both patients with and without spinal dysraphism [19, 20]. Long-term outcomes like this after the MOMS trial are extremely limited and should be used to guide preoperative decision making as well as lifelong management of spinal cysts including appropriate imaging surveillance guidelines and surgical management strategies.

Limitations of this study include generalizability given this is a single-center cohort and limitations inherent to a retrospective chart review including selection bias and limitations of information from the electronic medical record. Future work should aggregate data across all major centers that perform fetal and postnatal myelomeningocele repairs and continue to analyze long-term outcomes of these patients as they enter until adulthood. Use of broad databases such as the Spina Bifida Registry or North American Fetal Therapy Network would allow for broader understandings of the incidence of inclusion cysts and their outcomes.

## Conclusion

Spinal cystic lesions are common in MMC and myeloschisis patients regardless of closure technique. The majority of these do not require surgery. We propose regular screening in both symptomatic and asymptomatic patients. Fetal repair is associated with a higher incidence of cyst and a higher proportion of cysts that require surgical intervention. However, these lesions can be safely surgically resected with maintenance of ambulation and urologic function.

## Data Availability

The data that support the findings of this study are not openly available due to reasons of sensitivity and are available from the corresponding author upon reasonable request.
